# The Influence of Microstructure on the Passive Layer Chemistry and Corrosion Resistance for Some Titanium-Based Alloys

**DOI:** 10.3390/ma12081233

**Published:** 2019-04-15

**Authors:** Nader El-Bagoury, Sameh I. Ahmed, Ola Ahmed Abu Ali, Shimaa El-Hadad, Ahmed M. Fallatah, G. A. M. Mersal, Mohamed M. Ibrahim, Joanna Wysocka, Jacek Ryl, Rabah Boukherroub, Mohammed A. Amin

**Affiliations:** 1 Department of Chemistry, Faculty of Science, Taif University, P.O. Box 888, Taif 21974, Saudi Arabia; nader_elbagoury@yahoo.com (N.E.-B.); o.abuali@tu.edu.sa (O.A.A.A.); a.fallatah.11@hotmail.com (A.M.F.); gamersal@yahoo.com (G.A.M.M.); ibrahim652001@yahoo.com (M.M.I.); 2 Department of Physics, Faculty of Science, Taif University, Hawiya 888, Saudi Arabia; sameh@sci.asu.edu.eg; 3 Department of Physics, Faculty of Science, Ain Shams University, Abbassia 11566, Cairo, Egypt; 4 Central Metallurgical Research and Development Institute, P.O. Box 87, Helwan, Cairo, Egypt; shimaamohamad901@yahoo.com; 5 Chemistry Department, Faculty of Science, South Valley University, Qena 83523, Egypt; 6 Chemistry Department, Faculty of Science, Kafrelsheikh University, Kafr El-Sheikh 33516, Egypt; 7 Department of Electrochemistry, Corrosion and Materials Engineering, Chemical Faculty, Gdansk University of Technology, Narutowicza 11/12, 80-233 Gdansk, Poland; joanna.wer.wysocka@gmail.com; 8 Univ. Lille, CNRS, Centrale Lille, ISEN, Univ. Valenciennes, UMR 8520-IEMN, F-59000 Lille, France; rabah.boukherroub@univ-lille.fr; 9 Department of Chemistry, Faculty of Science, Ain Shams University, Abbassia 11566, Cairo, Egypt

**Keywords:** titanium-based alloys, microstructure, passivity breakdown, pitting corrosion

## Abstract

The effect of microstructure and chemistry on the kinetics of passive layer growth and passivity breakdown of some Ti-based alloys, namely Ti-6Al-4V, Ti-6Al-7Nb and TC21 alloys, was studied. The rate of pitting corrosion was evaluated using cyclic polarization measurements. Chronoamperometry was applied to assess the passive layer growth kinetics and breakdown. Microstructure influence on the uniform corrosion rate of these alloys was also investigated employing dynamic electrochemical impedance spectroscopy (DEIS). Corrosion studies were performed in 0.9% NaCl solution at 37 °C, and the obtained results were compared with ultrapure Ti (99.99%). The different phases of the microstructure were characterized by X-ray diffraction (XRD) and scanning electron microscopy (SEM). Chemical composition and chemistry of the corroded surfaces were studied using X-ray photoelectron spectroscopy (XPS) analysis. For all studied alloys, the microstructure consisted of α matrix, which was strengthened by β phase. The highest and the lowest values of the β phase’s volume fraction were recorded for TC21 and Ti-Al-Nb alloys, respectively. The susceptibility of the investigated alloys toward pitting corrosion was enhanced following the sequence: Ti-6Al-7Nb < Ti-6Al-4V << TC21. Ti-6Al-7Nb alloy recorded the lowest pitting corrosion resistance (*R*_pit_) among studied alloys, approaching that of pure Ti. The obvious changes in the microstructure of these alloys, together with XPS findings, were adopted to interpret the pronounced variation in the corrosion behavior of these materials.

## 1. Introduction

Titanium and its alloys are widely used in many industrial applications, because of their highly desirable properties, including very good mechanical properties, excellent corrosion and erosion resistance, and favorable strength to weight ratio [1]. In fact, titanium and its alloys have experienced increased use in the past years as biomaterials, because of their superior biocompatibility, high resistance to localized and generalized corrosion, and their good mechanical properties (fatigue resistance) [2]. Among all titanium and its alloys, the commonly used materials in biomedical area are commercially pure titanium (cpTi) and its (α + β) Ti6-Al4-V alloy [3,4,5].

Next to biomedical applications, aerospace sector has dominated titanium use, instead of heavy steel components, in the fabrication of crucial and decisive systems such as airfoils and airframes [6,7,8,9]. About 50% of titanium used in the aerospace industry is the (α + β) Ti-6Al-4V alloy. This alloy possesses a perfect combination of operational and technological properties [10,11]. Titanium alloys have also found widespread applications in a variety of fields such as in chemical and petrochemical sectors due to their excellent corrosion resistance [12]. The outstanding characteristics (such as high specific strength, high fatigue strength, good corrosion resistance, etc.) of the titanium alloys (particularly Ti-6Al-4V) are attributed to a very stable native oxide film (1.5–10 nm) formed on the Ti and Ti-alloy surface upon exposure to atmosphere and/or aqueous environments [13,14]. However, this thin oxide layer can be damaged and thus strongly impacts the bioactivity and other characteristics of the material. To improve the performance of Ti and Ti-alloys for biomedical and aerospace applications, oxidation (anodization) has been applied as a successful approach to improve the material properties [15,16].

The microstructure, formed during various processing methods, is found to greatly influence the mechanical properties of titanium alloys [17]. The microstructure type (bimodal, lamellar and equiaxed) affects the mechanical properties of Ti based alloys [18]. Even though the corrosion of Ti-alloys in different environments has previously beenstudied [19,20,21,22,23], to the best of our knowledge, the literature contains no reports onthe passive layer growth kinetics and breakdown, and subsequent initiation and propagation of pitting corrosion over the surfaces of Ti-6Al-7Nb, Ti-6Al-4V, and TC21 alloys. In this context, the mainobjective of our studywas to assess the effect of microstructure changes of tested alloys on their surface morphology and chemistry using different techniques such as scanning electron microscopy with electron dispersive X-ray spectroscopy (SEM/EDX), X-ray diffraction (XRD), and X-ray Photoelectron Spectroscopy (XPS). The influence of microstructure changes on the anodic behavior and passive layer growth kinetics and breakdown was also investigated. The corrosion resistances were compared using potentiodynamic polarization and impedance spectroscopy tools. All measurements were conducted in 0.9% NaCl solution at 37 °C.

## 2. Materials and Methods

The working electrodes investigated in this study consisted of three Ti-based alloys, namely Ti-6Al-4V, Ti-6Al-7Nb and TC21; their chemical compositions are presented in Table 1. The as-received titanium alloy samples were prepared by melting in a 500 kg vacuum induction furnace to obtain billets. These billets were then forged and machined into 10 mm diameter bars. The microstructures of these alloys werestudied by Meiji optical microscope (Meiji Techno Co., Ltd., Chikumazawa, Japan) fitted with a digital camera (Meiji Techno Co., Ltd., Chikumazawa, Japan). JEOL JSM5410 and Hitachi S-3400N scanning electron microscopes (LxRay Co., Ltd., Hyogo, Japan) (SEM) were also used for microstructure studies. For this purpose, the specimens were prepared following ASTM E3-11 standard metallographic procedures, and then etched in a mixture of 5 mL HNO_3_, 10 mL HF and 85 mL H_2_O. The alloys were machined in the form of rods to perform electrochemical measurements. These rods were mounted in a polyester resin offering an active cross-sectional area of ~0.2 cm^2^. Prior to conducting any electrochemical analysis, the surface of the working electrode was cleaned and polished using a silicon carbide paper (600-grit) installed on a polishing machine (Minitech 233). The surface was then washed copiouslywith distilled water and rinsed with absolute ethanol (SIGMA-ALDRICH, Steinheim, Germany).

Electrochemical measurements were conducted in a standard, double-walled electrochemical cell (Princeton Applied Research, USA) with an inner volume capacity of 200 mL. Temperature of the test solution was maintained constant at the desired value by means of a temperature-controlled water bath (FP40-MA Refrigerated/Heating Circulator) (JULABO GmbH, Seelbach, Germany). The water, after being adjusted to 37 ± 0.1 °C, wasallowed to circulate through external jacket of the cell. The cover of the electrochemical cell had five openings with different sizes. Such openings were designed to be fitted to the working electrode, counter electrode (a long, coiled platinum wire), reference electrode (KCl-saturated calomel electrode (SCE)), a thermometer and a gas inlet/outlet for gas release. The reference electrode was placed in a Luggin capillary, the tip of which was adjusted to be close to the working electrode to minimize iR drop. The cell was connected to a Potentiostat (Autolab PGSTAT30) (Metrohm, Herisau, Switzerland). The test solution was a normal saline (0.9% NaCl). A Millipore Milli-Q water system (Merck Millipore, MA, USA) (18.2 MΩ cm) was used to freshly prepare the saline solution. The salt was of analytical grade and purchased from Sigma-Aldrich (Steinheim, Germany).

Linear sweep voltammetry (LSV), Tafel plots, and Electrochemical Impedance Spectroscopy (EIS) techniques were applied to investigate the uniform corrosion characteristics of the studied alloys. The susceptibility of these alloys to passivity breakdown was evaluated via conducting potentiodynamic polarization measurements. Uniform corrosion measurements were started by stabilizing the working electrode at the rest potential for 2 h, followed by conducting EIS measurements at the respective corrosion potential (*E_corr_*) every day for a week of exposure in 0.9% NaCl solution at 37 °C, covering a wide frequency range (100 kHz–10 mHz), with 15 mV perturbation amplitude. Uniform corrosion study was assessed by constructing Tafel plots via sweeping the electrode potential around the Tafel potential (*E* = *E*_corr_ ± 250 mV), applying a sweep rate of 1.0 mV s^−1^. After that, the electrode was removed from the cell (which was cleaned properly and re-filled with a new fresh test solution), cleaned and polished up to the mirror finish, as described above, and then inserted in the cell for cyclic polarization measurements. Chronoamperometry (CA) technique was also applied using a new set of cleaned and polished electrodes submerged in a cleaned cell filled with a new fresh solution. 

Prior to performing cyclic polarization measurements, the working electrode was allowed to stabilize at rest potential for 2 h, then swept linearly, at a sweep rate of 1.0 mV s^−1^, starting from a cathodic potential of −2.0 V to +8.0 V vs.SCE. The potential sweep was then reversed back with the same sweep rate to reach the start point again, thus forming one complete cycle. To conduct chronoamperometry (current vs. time) measurements, a two-step route was applied. The working electrode was first held at a starting cathodic potential of −2.0 V vs. SCE for 60 s, and then polarized towards the anodic direction at a sweep rate of 1.0 mV s^−1^ untilthe required anodic potential (*E*_a_). Finally, the anodic current was measured versus time (5.0 min) by holding the working electrode at *E*_a_. To ensure reproducibility, each run was repeated at least three times, where mean values of the various electrochemical parameters and their standard deviations were calculated and reported.

The XRD diffraction patterns were collected for the bulk samples using a SmartLab SE (Rigaku Americas Corporation, Oxford, MS, USA) X-ray diffractometer with Cu Kα (λ = 1.54056 Å) operated at 40 kV and 40 mA. The scanning speed was 0.2°/min and the scanning angle ranged from 20° to 100° in *2θ*. Energy dispersive X-ray spectroscopy (EDS) measurements were utilized to determine microstructural composition of investigated alloys as well asevaluate changes in chemical composition as a result of exposure to corrosive media. S-3400N SEM (Hitachi, Tokyo, Japan) was equipped with an UltraDry detector from ThermoFisher Scientific (Waltham, MA, USA). High-resolution X-ray photoelectron spectroscopy (XPS) studies were carried out on an Escalab 250 Xi from Thermofisher Scientific (Waltham, MA, USA), equipped with Al Kα source. Pass energy was 20 eV and the spot size diameter was 650 μm. Charge compensation was controlled through the low-energy electron and low energy Ar^+^ ions emission by means of a flood gun (emission current: 150 μA, beam voltage: 2.1 V, filament current: 3.5 A). Avantage software (Thermofisher Scientific, Waltham, MA, USA) was used for deconvolution purposes.

## 3. Results and Discussion

### 3.1. Microstructure Investigation

**Figure 1:**
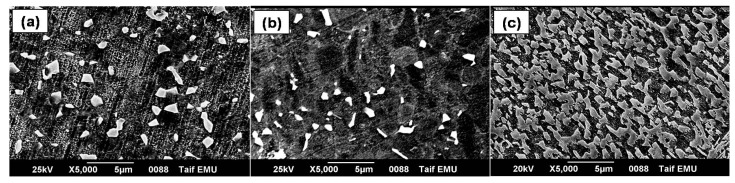
Microstructure of the three investigated Ti alloys: (**a**) Ti-Al-V; (**b**) Ti-Al-Nb; and (**c**) TC21.

Based on the morphology of α phase, the microstructure of titanium alloys can be classified into equiaxed, lamellar and bi-modal microstructures [24]. The microstructure of Ti-based alloys can be controlled based on their chemical composition, i.e., based on the balance between the α phase stabilizing elements, such as Al, Sn and O, and the forming β phase elements such asV, Mo and Nb [25]. As shown in Figure 1, the microstructure of all studied titanium alloys consisted of bimodal structure of α/β phases. The initial microstructure of Ti-Al-V and Ti-Al-Nb alloys in as-received (forged) state was represented by equiaxed grains of primary αphase (dark), as well as β-transformed structure (light), as shown in Figure 1. The β phase formed in the microstructure of both alloys was globular in shape, but seemed larger in size in Ti-Al-V alloy than in Ti-Al-V alloy. The particle size of β phase in Al-Ti-V alloy was about 0.5–1.5 μm, while its size in Ti-Al-Nb alloy was slightly lower (about 0.25–1 μm), as depicted in Figure 1a,b.

**Figure 2:**
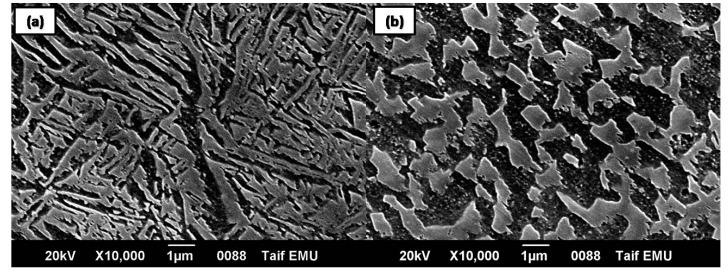
Morphology of β phase in TC21 alloy: (**a**) acicular-like structure; and (**b**) blocky shaped structure.

Similar to Ti-Al-V and Ti-Al-Nb alloys, the microstructure of TC21 alloy (Figure 1c) contained α and β phases, but displayed different morphologies and volume fractions. The TC21 alloy’s β phase consisted of two shapes:an acicular-like structure (Figure 2a) and a blocky shape (Figure 2b). The thickness of the acicular β phase in the TC21 alloy’s microstructure ranged from around 0.2 to 0.6 μm, while the size extent of the blocky β phase was about 0.75–1.5 μm. Moreover, the volume fraction of β phase in the microstructure of TC21 alloy was higher than that in the Ti-Al-V and Ti-Al-Nb alloys’ microstructure, as depicted in Figure 1.

Table 2 illustrates the volume fraction of α and β phases in the microstructure of the studied titanium alloys. The microstructure of pure Ti has the highest volume fraction of the α phase (~100%) and the lowest volume fraction of β phase (~0.0%). The presence of Al (α-phase stabilizer) and V (β-phase stabilizer) as alloying elements in the chemical composition of Ti-Al-V alloy influenced the volume fraction of α and β phases. The values of the volume fractions of α and β phases (Table 2) in the microstructure of Ti-Al-V alloy were 65% and 35%, respectively. Replacing V with Nb, yielding Ti-Al-Nb alloy, resulted in an obvious enhancement in the volume fraction of α phase (increased to 77%) at the expense of that of the β phase, which decreased to 23%, as shown in Table 2. The volume fraction of both phases in the microstructure of TC21 alloy wasalso altered, probably due to the mutual combination of the alloying elements of that alloy (cf. Table 1). The volume fractions of α and β phases in the microstructure of TC21 alloy recorded almost equal values: 48% for α phase and 52% for β phase (Table 2).

To further assess the influence of chemical composition on the microstructure and volume fraction of α and β phases, [Al]_eq_ and [Mo]_eq_ were calculated, where [Al]_eq_ and [Mo]_eq_ represent the alloying elements from α and β phases, respectively [5,26]. Table 3 illustrates the calculated values of [Al]_eq_ and [Mo]_eq_ for the tested Ti-based alloys, following Equations (1) and (2) [5,26].

(1) $${\left[Al\right]}_{eq.}=\left[Al\right]+0.33\left[Sn\right]+0.17\left[Zr\right]+10\left[O+C+2N\right]$$

(2) $${\left[Mo\right]}_{eq.}=\left[Mo\right]+0.2\left[Ta\right]+0.28\left[Nb\right]+0.4\left[W\right]+0.67\left[V\right]+1.25\left[Cr\right]+1.25\left[Ni\right]+1.7\left[Mn\right]+1.7\left[Co\right]+2.5\left[Fe\right]$$

As shown in Table 3, Ti-6Al-7Nb and TC21 alloys recorded the highest value (8.59) of [Al]_eq_, while the lowest value (8.05) was measured for the Ti-6Al-4V alloy. Additionally, Ti-6Al-7Nb alloy achieved the maximum value of [Mo]_eq_, 3.94, whileTC21 alloy recorded 1.71. Table 3 also depicts the ratio [Al]_eq_/[Mo]_eq_ for the tested alloys. Ti-6Al-7Nb alloy displayed the maximum ratio, 3.94, while a minimum ratio of 1.71 was determined for the TC21 alloy. The results shown in Table 3 agree well with those in Table 2. The calculated ([Al]_eq_/[Mo]_eq_) and (α/β) ratios weremaximum in case of Ti-6Al-7Nb alloy, and minimum for the TC21 alloy.

The chemical composition of both phases in all microstructures of the investigated alloys was analyzed using the EDS unit attached to SEM. The averaged chemical composition (wt. %) for each investigated alloy is displayed in Table 4 and Table 5. It was evident that the Ti, Al, Sn and Zr elements tended to more segregate to α phase than to β phase [27]. However, V, Nb, Cr and Mo were β forming elements [28], meaning that higher ratios of these elements werefound in β phase rather than in α phase.The detailed EDS linescan/map analyses are discussed in the Supplementary Materials (Figures S1–S3).

### 3.2. X-ray Diffraction Studies

**Figure 3:**
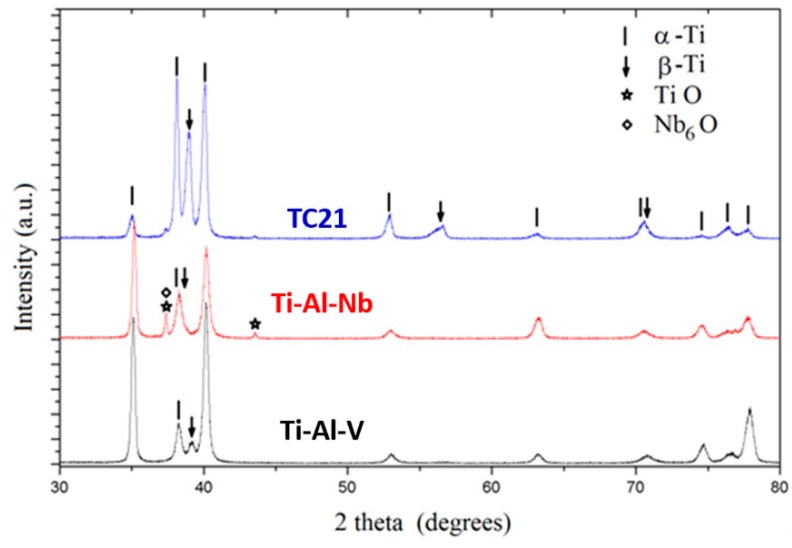
XRD diffraction patterns recorded for the Ti-6Al-4V, Ti-6Al-7Nb and TC21 samples.

Phase identification was performed by X-ray diffraction (XRD) patterns to define the phases comprising each alloy sample. The diffraction patterns recorded for the studied alloys are compared in Figure 3. The phases were identified by matching the characteristic peaks with the Joint Committee on Powder Diffraction Standards (JCPDS) files [29]. The phases α-Ti (JCPDS#00-044-1294) andβ-Ti (JCPDS#00-044-1288) were common and dominated the composition of the three studied alloys. The Ti-Al-V and TC21 alloys were found to contain solely α-Ti and β-Ti phases, respectively. On the other hand, Ti-Al-Nb alloy contained some Ti and Nb oxides, TiO (JCPDS#00-008-0117) and Nb_6_O (JCPDS#00-015-0258).

An effective procedure for the simultaneous refinement of structural and microstructural parameters based on the integration of Fourier analysis for broadened peaks in the Rietveld method was first proposed by Lutterutti et al. [30] and is implemented in the Maud program [31]. Consequently, weight percent (wt.%), lattice parameters, isotropic crystallite size (D) and r.m.smicrostrain (µε) were regarded as fitting parameters in the Rietveld adjustments and refined simultaneously. The structural information for all the refined phases was obtained from the Inorganic Crystal Structure Database (ICSD) [32]. The results obtained for the structural and microstructural analysis are summarized in Table 6 for all alloys. It is worth mentioning that all studied alloys were characterized with considerable degree of preferred orientation, which strongly modified the relative intensities of the Bragg reflections, especially for α-Ti and β-Ti phases. The MAUD program also incorporates correction for preferred orientation [33,34] in the Rietveld adjustments to obtain the best fitting parameters.

**Figure 4:**
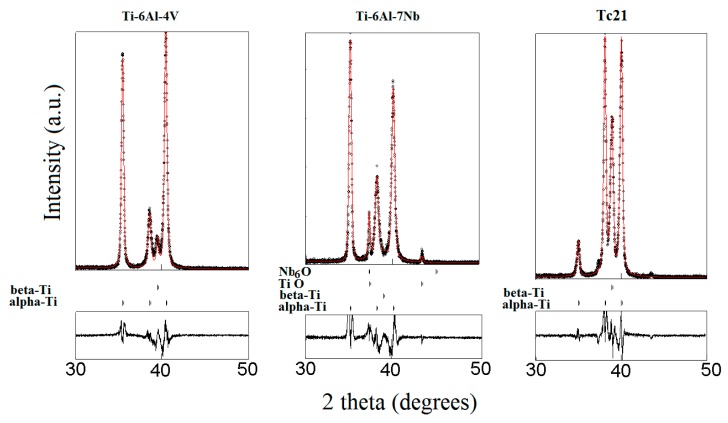
The calculated (red line) and recorded (black dots) diffraction patterns for the three alloys as obtained from the Rietveld adjustments using the MAUD program; the positions of the Bragg reflections of each phase and the difference between the calculated and observed patterns are also presented at the bottom.

The calculated diffraction patterns from the Rietveld adjustment are plotted with the observed ones for the three alloys in Figure 4. The average R-values obtained for the refinements were about Rwp (%) = 24–27 and Rb (%) = 15–20. The simultaneous refinements of both structural and microstructural parameters produced good matching of the calculated to observed profiles of diffracted intensities. In addition, the incorporation of the preferred orientation models enabled accounting for the variations of the peak intensities of α and β-Ti phases.

In the Rietveld adjustment of the Ti-6Al-4V alloy, the hcp α-Ti (Space group $P6_{3}/mmc$) together with the bcc β-Ti (Space group $Im\bar{3}m$) dominated the composition of the alloy. In the second alloy, Ti-6Al-7Nb, the formation of some TiO (Space group $Fm\bar{3}m$) and Nb_6_O (Space group P42cm) was observed and they formed larger crystallites than those formed in the Ti phases. The total weight percent of those oxide phases was less than 10% (Table 6). For the third alloy, TC21, only α- and β-Ti phases were observed in the XRD patterns. No oxide phases were detected due to the slight oxygen content of this alloy. Nevertheless, there weresome mismatches between the wt% values obtained from the Rietveld adjustments and the corresponding wt% values determined with other techniques. This was attributed to the behavior of the preferred orientation of the α-Ti phase observed for the reflection (100), which was relatively stronger for the Ti-6Al-4V and Ti-6Al-7Nb alloys than in the TC21 alloy.

As shown in Table 6, the last two alloys, Ti-6Al-7Nb and TC21, contained relatively higher portions of β-Ti than α-Ti in contrast to the first alloy, Ti-6Al-4V, which hadα-Ti content higher than β-Ti. As known from the literature, Al is an α-stabilizing while V, Nb, Mo and Fe are β-stabilizing elements. Nevertheless, the results indicate that Nb, Mo and Fe hadstronger capabilities to stabilize β-Ti phase than V. These findings corroborate microstructural studies (cf. Section 3.1).

### 3.3. Electrochemical Measurements

#### 3.3.1. Uniform Corrosion Studies

**Figure 5:**
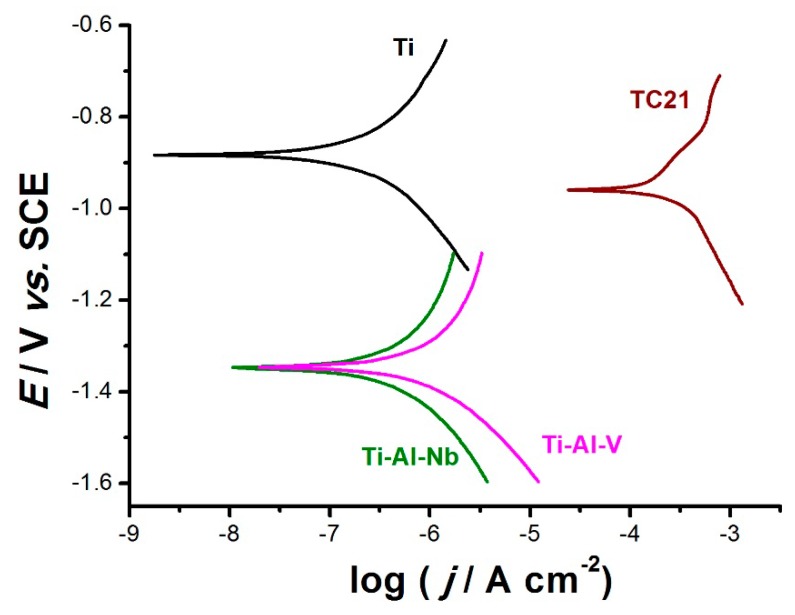
Cathodic and anodic polarization curves recorded for the three tested alloys in comparison with pure Ti, after seven days of exposure in 0.9% NaCl solution at a scan rate of 0.5 mV s^−1^ at 37 °C.

Figure 5 illustrates the cathodic and anodic polarization curves for the studied alloys in comparison with pure Ti, after seven days of immersion in 0.9% NaCl solution at 37 °C. As shown in Figure 5, among the studied alloys, TC21 alloy exhibited the lowest cathodic and anodic overpotentials, corresponding to enhanced corrosion rate. On the contrary, Ti-6Al-7Nb alloy displayed the highest overpotentials, close to that of Ti, for both the cathodic and anodic processes, thus referring to its highest corrosion resistance.

Indeed, Tafel slopes and respective calculated uniform corrosion rates for a metal covered with a semi-conductive passive film raisedstrong doubts, despite the utilization of the Tafel extrapolation method to determine corrosion rates for Ti and some Ti-based alloys [35,36]. In addition, in Figure 5, the polarization curves do not display the expected log/linear Tafel behavior. This was clear in both the anodic and cathodic branches of alloys Ti-6Al-4V and Ti-6Al-7Nb and as well as in the anodic branches of Ti and TC21 alloy, which exhibited some sort of curvature over the complete applied potential range. This in turn made evaluation of Tafel slopes by Tafel extrapolation method, and hence corrosion rates, inaccurate [37,38,39]. There is, therefore, an uncertainty and source of error in the numerical values of Tafel slopes (β_a_ and β_c_), and possibly in the values of j_corr_. 

The curvature of the anodic branch mightbe attributed to the deposition of the corrosion products and/or passive film formation, as evidenced from XPS studies. With respect to the cathodic branch, since the solution was stationary, diffusion of the electrochemically active species was slow, and concentration polarization couldact to shorten the cathodic linear Tafel region. In the extreme case, linearity mightdisappear altogether, with the cathodic reaction proceeding under combined activation and diffusion control at E_corr_ [37,38,39]. This counteracted the validity of the Tafel extrapolation method for measuring uniform corrosion rates, which was successfully applied for the charge transfer controlled processes.

**Figure 6:**
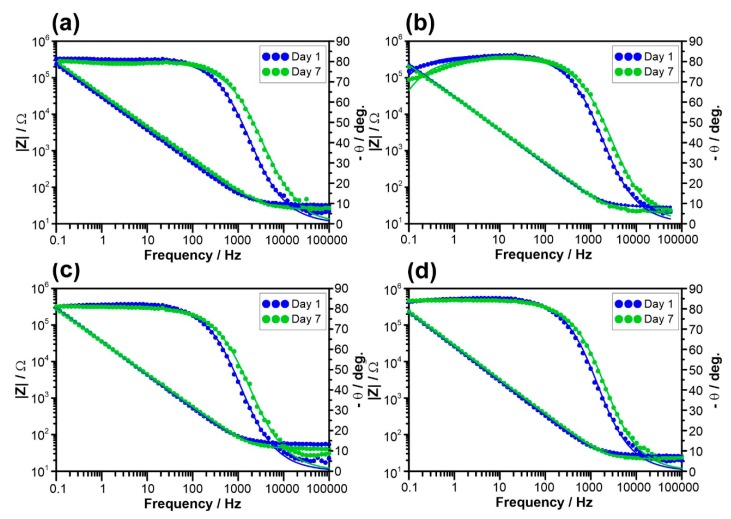
Bode impedance plots on Days 2 and 7 of exposure, recorded for: (**a**) pure Ti; and three tested alloys: (**b**) TC21; (**c**) Ti-6Al-4V; and (**d**) Ti-6Al-7Nb. Studies performed at E_corr_ in 0.9%NaCl solution at 37 °C. Points represent experimental results while the solid line was calculated based on R(QR) EEC.

EIS measurements were also conducted at the respective E_corr_ during sample’s exposure in 0.9% NaCl solution at 37 °C to confirm the polarization data and to assess the kinetics of the uniform corrosion process on alloy surface. The measurements were carried day-by-day allowing for monitoring of uniform corrosion susceptibility [40,41,42]. Figure 6 displays the impedance plots in Bode projection, recorded for the studied alloys during the second and the last day of exposure. Pure Ti (99.99%) was also included for comparison (see Figure 6a). The impedance spectra recorded on Day 1 (after initial 120 min of conditioning) were highly scattered due to non-stationary conditions at the metal/electrolyte interface, which is a common problem in EIS measurements. This issue became negligible after a few hours of exposure. For this reason, results recorded on Days 2–7 were considered for further analysis. All the impedance plots exhibited a single time constant (capacitive loop). The overall corrosion resistance of each investigated alloy was very high; the impedance modulus |Z| linearly increasedwith frequency decrease, reaching over 10^5^ at 0.1 Hz. For each studied alloy, inclination of the phase angle θ was shifted towards lower frequencies on Day 7 of exposure, testifying tothe decreased corrosion process kinetics.

An electric equivalent circuit (EEC) was proposed to analyze the impedance results. When defining the adequate EEC, one must consider whether to include the space charge layer resulting from the semi-conductive nature of titanium oxides. In his studies, Blackwood concluded that thickness of the space charge layer is considerably less than the oxide film itself in the open circuit conditions [43]. On the other hand, its dominant influence was observed under anodic polarization conditions [43,44,45,46]. The impedance measurements of titanium oxide films investigated within this manuscript were studied under open circuit conditions, thus the parallel resistance and CPE represented primarily the dielectric properties of the passive layer. Due to absence of additional time constants in the analyzed frequency rage, a simple Randles circuit was proposed with constant phase element (CPE) selected instead of capacitance to account for the dispersion of the time-constant. The parallel resistance represents the sum of charge-transfer limiting effects through the metal/electrolyte interface, dominated by the passive layer resistance R_F_ [47]. The aforementioned time-constant dispersion originated from thepresence of the charge space layer in the semi-conductive film, thesurface distribution of the time-constant due to the geometric heterogeneity (pits, scratches, and porosity), and diversified surface electric properties due to adsorption processes of passive layer breakdown [48]. 

The CPE impedance Z_CPE_ = (Q(jω)^n^)^−1^ represents a capacitor with capacitance 1/Q for a homogeneous surface n→ 1. Thus, it is often believed that CPE component n is the heterogeneity factor and its variation can be monitored. CPE describes quasi-capacitive behavior of the passive layer. The effective capacitance C_eff_ can be calculated based on CPE using Hirschorn’s model for surface distribution of time constants [49]. The EEC can be schematically written as R_S_(QR_F_), where R_S_ is electrolyte resistance. The aforementioned single time-constant EEC covers all the applied frequency range. The fitting quality is represented by solid lines in Figure 6a–d.

**Figure 7:**
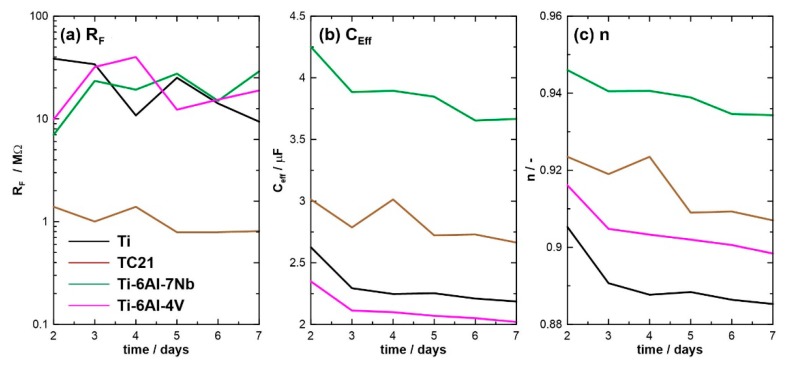
Monitoring of (**a**) passive layer resistance R_F_, (**b**) effective capacitance C_eff_ and (**c**) CPE exponent n calculated on the base of R_S_ (QR_F_) EEC for each investigated alloy. The one-week exposure was carried out in 0.9% NaCl solution at 37 °C.

Figure 7 depicts the electric parameters obtained on the base of R_S_(QR_F_) EEC and their changes during the one-week exposure. The higher was the R_F_, the lower was the corrosion current density, offering an easy comparison in uniform corrosion resistance of investigated alloys (see Figure 7a). Each investigated alloy was characterized with very high resistance, order of MΩ, owing to a presence of a passive layer tightly covering the metal surface. Nevertheless, for TC21 alloy, R_F_value wasone order of magnitude lower and slowly but consistently decreased throughout the exposure, revealing its lower corrosion resistance and corroborating DC electrochemical studies.

The analysis of constant phase element (CPE) alloweddrawing conclusions onthe passive layer homogeneity. The effective capacitance C_eff_, calculated using the surface distribution model, was similar for allinvestigated alloys, falling in a range between 2 and 5 μF. The differentiation may result from differences in passive layer thickness d and to some extent from relative permittivity of alloying additives and their oxides ε_r_ according to: C = ε_0_ε_r_d/A, where ε_0_ is the absolute permittivity and A is the electrochemically active surface area. A steady decrease of C_eff_ was attributed to an increase of passive layer thickness, denouncing further passivation of metal in investigated electrolytic conditions. The presence of stable corrosion pits would be visible in theform of rapid increase in C_eff_ [49,50] (likely observed in TC21 alloy on Day 4).

**Figure 8:**
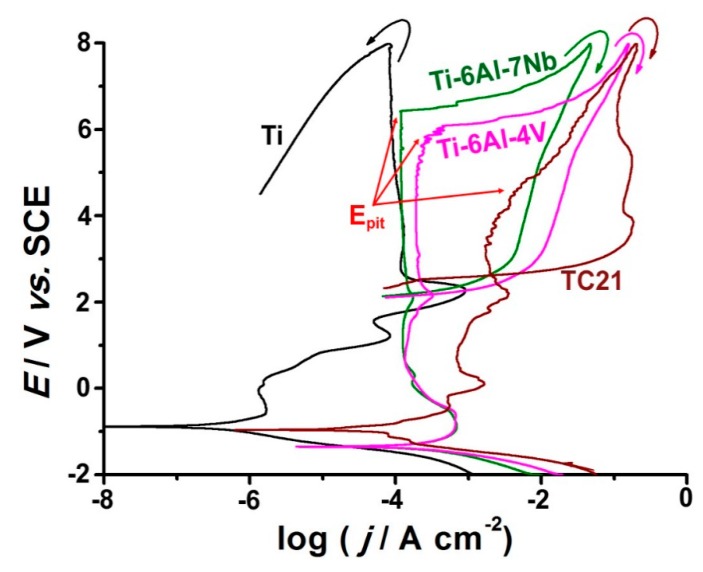
Cyclic polarization curves recorded for the studied alloys in 0.9% NaCl solutions at a scan rate of 1.0 mV s^−1^ at 37 °C.

The initial value of CPE exponent n depends on factors such as surface phase distribution and geometric defects remaining after polishing. Its decrease throughout the exposure in corrosive electrolyte reflected the appearance of heterogeneities on analyzed sample surface, which in this case was primarily associated with initial phases of corrosion pits formation (see Figure 8c). This effect was clearly seen in SEM micrographs (see below). Notably, the value of n factor of Ti-6Al-7Nb alloy was both the highest and the least affected by exposure in corrosive media. The aforementioned observation indicated high surface homogeneity, which may be the reason behind outstanding corrosion resistance of this alloy.

#### 3.3.2. Cyclic Polarization Measurements

Figure 8 shows typical cyclic polarization curves recorded for the studied alloys between −2.0 V and + 8.0 V (SCE). Measurements were conducted in 0.9% NaCl solution at a scan rate of 5.0 mV s^−1^ at 37 °C.

The polarization curve of TC21 alloy exhibited active dissolution near E_corr_, followed by an obvious enhancement in the anodic current with the applied potential due to thinning and weakening of the passive layer as a result of the aggressive attack of Cl^−^ anions. In addition, Ti-6Al-7Nb and Ti-6Al-4V alloys showed active dissolution near E_corr_, but to a much lower extent than TC21, and, in addition, tended to passivate with a very low current covering a wide range of potential. These findings reflect the weaker passivity of TC21 and its higher tendency to corrode in this solution than Ti-6Al-4V and Ti-6Al-7Nb alloys. On the contrary, as expected, the anodic polarization curve of pure Ti exhibited typical passivity near E_corr_, referring to its high corrosion resistance. 

Passivity of the studied alloys persisted up to reaching pitting potential (E_pit_). Remarkable changes occurred within the passive region at potentials exceeding E_pit_. These involved a sudden increase in corrosion current density and formation of a hysteresis loop on the reverse potential scan. These events werea clear sign ofpassivity breakdown, and initiation and propagation of pitting corrosion. Repassivation was only achieved when the reverse scan intersected the forward one within the passive region, below which the working electrode was immune to pitting [51].

A current intermission could be seen on the reverse scan of the three tested alloys. This current discontinuity aws quite clear on the reverse scan of the TC21 alloy, and couldbe observed for Ti-6Al-7Nb and Ti-6Al-4V alloys. We previously reported similar findings during pitting corrosion studies of Zn in nitrite solutions [52]. Recently, Zakeri et al. [53] explored the transition potential and the repassivation potential of AISI type 316 stainless steel in chloride containing media devoid of and containing 0.01 M thiosulfate.

Beyond pit transition potential, the rate of anodic dissolution was diffusion-controlled [51,52,53]. Such a current transient relationship, when satisfied, referred to an anodic diffusion control process [53]. On reversing the potential scan, the thickness of the salt (pitting corrosion product) film diminished. This decrease in salt film thickness enhanced with back scanning untila certain potential was reached, at which the cation concentration decreased below the saturated concentration. At this stage, salt precipitation was stopped, and the remaining metal salt film dissolved, making the bottom of pits free from salt film. This in turn established an ohmic/activation control (a linear decrease of current density with potential) regime. 

Ti-6Al-7Nb alloy’s passivity seemed stronger and more stable that of the Ti-6Al-4V alloy. The latter was characterized by a higher j_pass_, which enhanced with potential untilits E_pit_, which attained ~50 mV vs. SCE before that of the former. In addition, the pits existing on the surface of Ti-6Al-4V alloy were much more difficult to repassivate than those on the surface of Ti-6Al-7Nb alloy, as the hysteresis loop of the former was much larger than that of the later. 

Another important pitting corrosion controlling electrochemical parameter is the pitting corrosion resistance R_pit_ = |E_corr_ − E_pit_|, which defines the resistance against the nucleation of new pits [38]. Referring to Figure 8, it is clear that R_pit_ increased following the order: TC21 << Ti-6Al-4V < Ti-6Al-7Nb. The resistance against growth of the pits also controlled the susceptibility toward pitting corrosion. A specific routine of the software (Autolab frequency response analyzer (FRA) coupled to an Autolab PGSTAT30 potentiostat/galvanostat with FRA2 module) (Metrohm, Herisau, Switzerland.) was used to calculate the areas of the hysteresis loops, related to the charge consumed during the growth of such already formed pits. Again, the hysteresis loop of the TC21 alloy recorded the highest area (charge consumed) among the studied alloys, while the lowest value of the hysteresis loop charge consumed during was measured for Ti-6Al-7Nb alloy. Thus, the resistance against the growth of pre-existing pits was ranked as: Ti-6Al-7Nb > Ti-6Al-4V >> TC21. These findings mean that replacing V by Nb in Ti-6Al-4V alloy promoted alloy’s repassivation, thus enhancing its pitting corrosion resistance.

#### 3.3.3. Chronoamperometry Measurements

**Figure 9:**
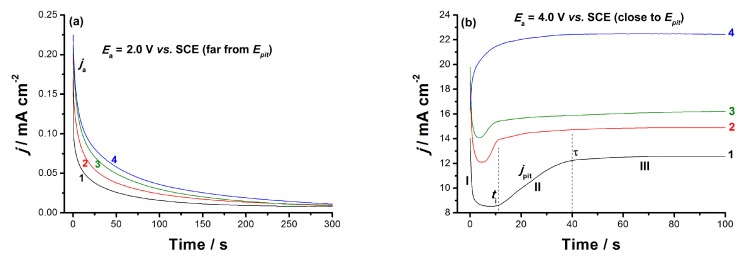
Chronoamperometry (current–time) curves recorded for the studied solder alloys in 0.9% NaCl solution at applied anodic potentials of 2.0 V (**a**) and 4.0 V (**b**) vs. SCE at 37 °C: (1) pure Ti; (2) Ti-6Al-7Nb; (3) Ti-6Al-4V; and (4) TC21.

**Figure 10:**
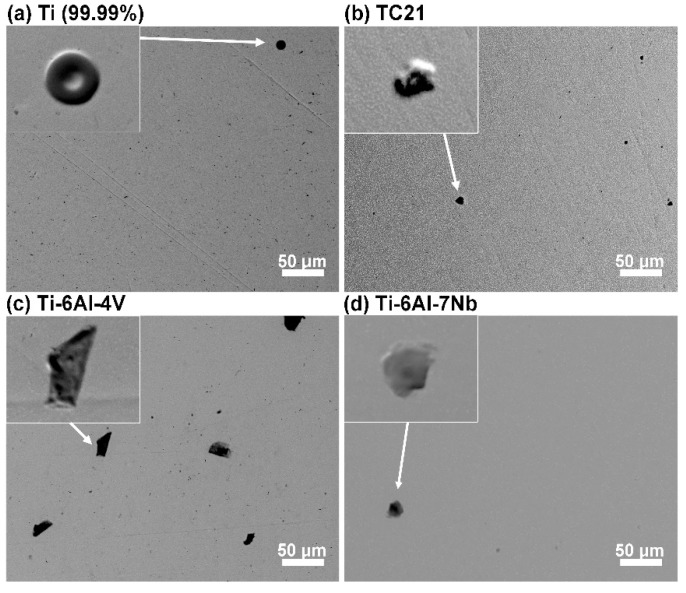
SEM micrographs taken in secondary electron mode for each investigated sample: (**a**) pure Ti as a reference; (**b**) TC21 alloy; (**c**) Ti-6Al-4V; and (**d**) Ti-6Al-7Nb at the end of one-week exposure in 0.9% NaCl at 37 °C. Magnification: × 500. In the inset, back-scatter electron topography mode images of selected surface defects. Magnification: × 2000.

Chronoamperometry (*j*/*t*) measurements were also carried out to confirm the above results and gain more information about the influence of alloyed V and Nb on the passive layer growth kinetics and breakdown. Figure 9a and Figure 10b depict the *j*/*t* curves measured for the tested alloys at two different *E*_a_ values, far below and close to *E*_b_. Measurements were conducted in 0.9% NaCl solution at 37 °C. The profile of the obtained curves was found to vary according to the chemical composition of the studied alloy and the position of *E*_a_ versus *E*_pit_. When *E*_a_ was located far cathodic to *E*_pit_, a *j*/*t* profile with two stages was obtained, as shown in Figure 9a. During the first stage, the anodic current (*j*_a_) declined with a rate depending upon chemical composition of the tested alloy, denoting passive layer electroformation and growth [39]. This decay in current then reached a steady-state value (*j*_ss_), an almost constant passive current related to *j*_pass_ (cf. Figure 8), constituting the secondstage of the current. The constancy of *j*_ss_ originated from a balance between the rates of the passive layer growth (current builds up) and its dissolution (current decays) [54,55]. 

Further, Figure 9a demonstrates that the rate of *j*_a_ decay, and consequently the rate of passive layer growth, diminished upon alloying Ti with V and Nb. These results further confirm the influence of the alloying elements V and Nb, with V being more active than Nb, which, when added to Ti, weakened its passivity viadepassivation (destabilizing the passive oxide film through oxide film thinning/dissolution [56]). This in turn madethe passive film more susceptible to pitting. 

At an *E*_a_ value very close to *E*_pit_ (Figure 9b), the *j*/*t* curves with three different stages (I–III) were recorded. Similar results were previously obtained in our lab [55,57]. Stage I refersto the passive layer electroformation and growth, as its current fellwith time [54,55,57]. Stage I ended at a certain time (*t*_i_), the incubation time, where Stage I’s current reached its minimum value; *t*_i_ is defined as the time the adsorbed aggressive Cl^−^ anions must acquire to locally attack and subsequently remove the passive oxide film [54]. The magnitude of *t*_i_, more specifically its reciprocal value (1/*t*_i_), denotes the rate of pit initiation and growth [54,55], and measures the susceptibility of the oxide film to breakdown and initiate pit formation and growth. 

Stage II began at *t*_i_ and terminated at time *τ*, and its current aws termed *j*_pit_ (pit growth current density). *j*_pit_ increased from the moment just after *t*_i_ and continuedto grow until *τ*, suggesting that the pit formation and growth dominated over passivation during this stage. Ultimately, *j*_pit_ attaineda steady-state just after the time *τ*, denoting the onset of Stage III, and remained almost constant until the end of the run. The constancy of Stage III’s current was attributed to the hindrance of the current flow (*j*_pit_) through the pits sealed off by the pitting corrosion products formed during the events of Stage II, namely pit initiation and growth [55,57]. This hindrance in *j*_pit_ was balanced by a current increase due to metal dissolution, thus yielding an overall steady-state current. 

Close inspection of Figure 9 reveals that *j*_pit_ increased and *t*_i_ shortened, thus referring to accelerated pitting attack, in presence of alloyed V. These results again support the catalytic impact of alloyed V towards pitting corrosion

### 3.4. Surface Morphology and Composition

After one-week exposure, the investigated samples were reexamined using SEM to evaluate the susceptibility to pitting corrosion. This procedure was carried out after rinsing in ethanol using ultrasonic cleaner. The results of the analysis are exhibited in Figure 10. Defects started to appear on the surface of each analyzed sample throughout the exposure. The micrographs in the inset of Figure 10 were taken using back-scatter electrons (BSE) in topography mode. This allowed bringing out the geometry of aforementioned defects. As can be seen, each analyzed defect formed a bulge above the alloy’s surface, testifying to either repassivation once shallow corrosion pits formed or at an early stage of passive layer degradation. Ti-6Al-4V sample was characterized with both the highest amount and the largest defects, reaching 30 μm in diameter. On the other hand, the surface of pure Ti and Ti-6Al-7Nb appeared the most intact. No real corrosion pits were observed on the surface of either investigated alloy at the end of exposure in 0.9% NaCl solution at 37 °C, testifying tothe overall high pitting corrosion resistance.

Nevertheless, the passive layer must have weakened, hence it was possible for corrosion products to adsorb on the metal surface. EDS analysis was carried out on the defects observed on each investigated alloy to qualify their chemical constitution. The exemplary results, obtained for Ti-6Al-7Nb alloy, are summarized in Figure S4 (Supplementary Materials). The chemistry of defects observed for each investigated alloy was similar. The defects were primarily composed of carbon and oxygen, most likely forming metal carbonates typical for early pitting corrosion stages [58]. Small amount of chlorine was also found within defects. Its low amount was distorted by EDS depth of analysis ranging few microns.

**Figure 11:**
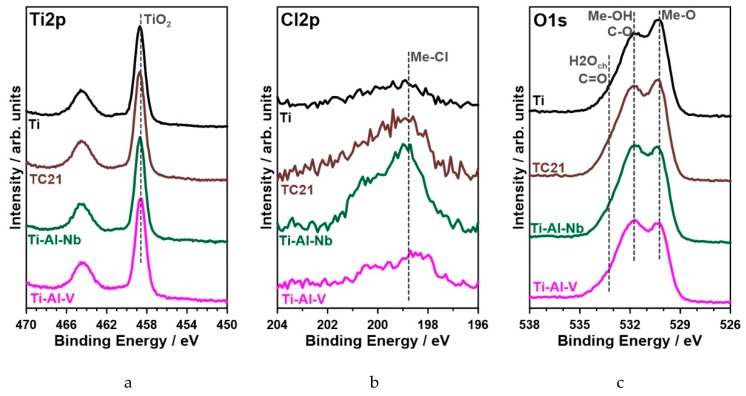
High-resolution XPS spectra recorded in (**a**) Ti_2p_, (**b**) Cl_2p_ and (**c**) O_1s_ energy range for each investigated alloy after seven days of exposure to 0.9% NaCl solution at 37 °C.

The chemistry of the passive layer in each examined case was composed primarily of titanium (IV) oxides, as verified by a strong recorded Ti_2p_ peak doublet, with Ti_2p3/2_ component located each time at 458.6 eV [48,59,60] (Figure 11). Furthermore, there was no sign of titanium oxides at lower oxidation states corroborating the aforementioned result (see Supplementary Materials, Figure S5). Besides the titanium, other alloying additives also tookpart in the passivation process. The strongest signal among the alloying additives was recorded for aluminum oxide Al_2_O_3_ (Al_2p3/2_ peak at 74.5 eV), ranging between 3.5 and 3.8 at.% for each sample [61,62]. The contribution of VO_2_ (V_2p3/2_ at 516.4 eV) in Ti-Al-V and Nb_2_O_5_ (Nb_3d5/2_ at 207.1 eV) in Ti-Al-Nb alloy did not exceed 0.7 at.% [60,63,64]. The passive film formed on the surface of TC21 alloy was naturally more complex. Besides TiO_2_, it wascomposed of Al_2_O_3_ (3.8 at.%), Nb_2_O_5_ (0.3 at.%), ZrO_2_ (0.4 at.%, Zr_3d5/2_ at 182.4 eV), Cr_2_O_3_ (0.8 at.%, Cr_2p3/2_ at 576.0 eV), SnO_2_ (0.1 at.%, Sn_3d3/2_ at 486.5 eV), MoO_3_ and MoO_2_ (0.2+0.2 at.%, Mo_3d5/2_ at 232.9 and 229.2 eV, respectively) [65,66,67,68].

The high-resolution spectra analysis carried out in the Cl_2p_ energy range confirmed the electrochemical and microscopic studies regarding chloride adsorption on the metal surface as a result of seven-day metal exposure to chloride-containing electrolyte. Full chemical analysis is summarized in Table 7. Metal chlorides were found on the surface of each investigated sample, which confirmed metal-chlorine bond formation, shownby a peak doublet: Cl_2p3/2_ at 198.9 eV [48,55,69]. Nevertheless, the amount of adsorbed chlorides Was nearly 2.5 times higher for the TC21 alloy than pure titanium. The chloride concentration obtained for highly resistant Ti-6Al-7Nb alloy was nearly on par withTi sample, and slightly smaller than in the case of Ti-6Al-4V. An interesting conclusion couldbe drawn based on O_1s_ peak analysis for each investigated sample. The spectra were conventionally deconvoluted into three components. Two dominant components located at 530.2 and 531.6 eV were ascribed to Me-O and Me-OH species, respectively. The second component intensity mightbe further influenced by the presence of C-O bonds in carbonates. Its formation may result from prolonged electrolyte exposure as well as adventitious carbon deposition due to air exposure [61,64]. The finding regarding carbonate adsorption on the metal surface wasfurther confirmed by a third O_1s_ component at 532.8 eV, typical for C=O bonds but also chemisorbed water molecules. For clarity purposes, the analysis excluded data recorded for carbon C_1s_, which was found in large amounts, up to 30 at.%, at binding energies corroborating adventitious carbon and carbonates. Importantly, the highest amount of the adsorbed carbonate species was found on Ti-Al-V sample surface, which is in very good agreement with SEM micrographs presented in Figure 10. The least amount of carbonate species was once more found on the surface of Ti sample.

## 4. Conclusions

The effect of microstructure on the uniform and pitting corrosion characteristics of Ti-Al-V, and Ti-Al-Nb alloys were studied. Pure Ti and TC21 alloy were included for comparison. Measurements were conducted in 0.9% NaCl solution at 37 °C employing various electrochemical techniques, and complemented with XRD and SEM/EDS analysis. The obtained results reveal that:

1. The microstructure of the investigated alloys consisted of α matrix, which was strengthened by β phase in all studied alloys except cp-Ti alloy.
2. The volume fraction of β phase in TC21 alloy was higher than in both Ti-Al-V and Ti-Al-Nb alloys. In comparison with other alloys, Ti-Al-Nb alloy had the lowest volume fraction of β phase.
3. Ti-Al-Nb alloy exhibitedthe highest corrosion resistance (lowest corrosion rate) among other alloys.
4. The addition of Nb alloying element at the expense of V in Ti-Al-V alloy decreased the volume fraction of β phase, which improvedthe corrosion resistance of Ti based alloy.
5. The polarization curve of TC21 alloy exhibited active dissolution near E_corr_, followed by enhanced anodic current with the applied potential, thus revealing weak passivity. On the contrary, Ti-6Al-7Nb and Ti-6Al-4V alloys recorded much lower dissolution rate near E_corr_ followed by a wide potential range of stable passivity.
6. Stable passivity of Ti-6Al-7Nb and Ti-6Al-4V alloys was translated into an obvious anodic (more noble) drift in their pitting potential (E_pit_) versus that of the TC21 alloy.
7. Chronoamperometry measurements, conducted at a fixed anodic potential, revealed that the rate of passivity breakdown of Ti-6Al-7Nb and Ti-6Al-4V alloys wasmuch lower than that of the TC21 alloy.
8. The XPS results reveal adsorption of chloride and carbonate species on the surface of the investigated alloys with the lowest amount recorded for cp-Ti and Ti-6Al-7Nb alloys, affecting the observed corrosion resistance.
9. Corrosion studies confirmed that the uniform and pitting corrosion rates increased following the sequence: Ti< Ti-6Al-7Nb < Ti-6Al-4V < TC21.

## Figures and Tables

**Table 1:** Chemical composition of investigated Ti alloys.

Alloy	Chemical Composition, wt %												
	Al	V	Nb	Sn	Zr	Mo	Cr	Si	Fe	C	N	O	Ti
Ti-6Al-4V	5.85	3.94	0.00	0.00	0.00	0.00	0.00	0.00	0.00	0.02	0.03	0.14	Bal.
Ti-6Al-7Nb	6.39	0.00	7.78	0.00	0.00	0.00	0.00	0.00	0.00	0.02	0.04	0.12	
TC21	5.89	0.00	2.41	2.51	1.59	2.27	1.58	0.067	0.05	0.01	0.01	0.13	

**Table 2:** Volume fraction of α and β phases in the investigated Ti based alloys.

Alloy	Volume Fraction, %		(α/ β) Ratio
	α Phase	β Phase	
Pure Ti	100	0	--
Ti-6Al-4V	65	35	1.86
Ti-6Al-7Nb	77	23	3.35
TC21	48	52	0.92

**Table 3:** and [Mo]_eq_ for the investigated alloys [26,27].

Alloy	[Al]_eq_	[Mo]_eq_	Ratio
Ti-6Al-4V	8.05	2.64	3.05
Ti-6Al-7Nb	8.59	2.18	3.94
TC21	8.59	5.04	1.71

**Table 4:** Chemical composition (wt %) of different phases in Ti-6Al-4V and Ti-6Al-7Nb alloys.

Phase	Ti-6Al-4V Alloy (wt %)			Ti-6Al-7Nb Alloy (wt %)		
	Al	V	Ti	Al	Nb	Ti
α	5.93	3.24	90.83	6.60	7.65	85.75
β	5.57	8.04	86.39	4.88	13.79	81.33

**Table 5:** Chemical composition (at %) of different phases in TC21 alloy.

Phase	Chemical Composition, at %						
	Al	Cr	Mo	Sn	Zr	Nb	Ti
α	6.38	1.08	1.82	2.45	1.95	1.98	84.34
β	6.21	1.71	2.62	2.24	1.11	2.39	83.72

**Table 6:** The structural and microstructural parameters of the three alloys obtained by the Rietveld adjustment of the XRD patterns. Wt. % is the weight percentage of each phase, a and c are the cell parameters of the crystal lattice, D is the average crystallite-size and ε is the lattice microstrain.

	**Ti-6Al-4V**			
	α-Ti ($P6_{3}/mmc$)	β-Ti ($Im\bar{3}m$)		
Wt. %	67 (5)	33 (5)		
a (Å)	2.9338 (1)	3.2353 (9)		
c (Å)	4.6780 (3)			
D (nm)	76 (3)	67 (4)		
ε	0.00232 (1)	0.00216 (4)		
	**Ti-6Al-7Nb**			
	α-Ti ($P6_{3}/mmc$)	β-Ti ($Im\bar{3}m$)	TiO ($Fm\bar{3}m$)	Nb6O (P42cm)
Wt. %	73 (6)	20 (5)	5 (2)	2 (1)
a (Å)	2.9397 (4)	3.2437 (5)	4.1567 (1)	3.3945 (6)
c (Å)	4.6969 (3)			3.249 (3)
D (nm)	67 (2)	53 (2)	79 (12)	100 (2)
ε	0.0028 (1)	0.024 (20)	0.0026 (1)	0.00010 (7)
	**TC21**			
	α-Ti ($P6_{3}/mmc$)	β-Ti ($Im\bar{3}m$)		
Wt. %	49 (9)	51 (9)		
a (Å)	2.9407 (7)	3.2521 (14)		
c (Å)	4.6925 (16)			
D (nm)	63 (2)	70 (3)		
ε	0.0021 (2)	0.0035 (3)		

**Table 7:** XPS deconvolution results carried out in Ti_2p_, Cl_2p_ and O_1s_ energy range after seven days of exposure to 0.9% NaCl solution at 37 °C (in at.%).

	Ti_2p_	Other Additives	O_1s_			Cl_2p_
	TiO_2_		Me-O	OH/CO	C=O/H_2_O	Me-Cl
BE/eV	458.6	*	530.2	531.6	532.8	198.9
Ti	21.3	--	37.5	26.2	13.1	1.9
Ti-6Al-4V	17.4	4.3	28.8	25.7	20.8	3.0
Ti-6Al-7Nb	17.1	4.4	31.1	27.0	18.2	2.2
TC21	17.2	5.8	31.2	25.1	16.1	4.6

## Supplementary Materials

The following are available online at https://www.mdpi.com/1996-1944/12/8/1233/s1. Figure S1—SEM image (a) and EDS spectrum (b) of phase in Ti-Al-V alloy; Figure S2—Line analysis of β phase in TiAlNb alloy; Figure S3—Microstructure of the Ti-Al-Nb alloy (a), and mapping of Ti (b), Al (c), and Nb (d) alloying elements. Figure S4—(a) SEM micrograph with marked areas for EDS analysis, (b) EDS examination at defect and at the surrounding, not corroded area. Figure S5—High-resolution Ti2p XPS spectra with the quality fit (light blue line) for each studied alloy: (a) pure Ti reference, (b) TC21, (c) Ti-6Al-4V, (d) Ti-6Al-7Nb.

## References

[1] de Assis S.L., Wolynec S., Costa I. (2006). Corrosion characterization of titanium alloys by electrochemical techniques. Electrochim. Acta.

[2] Geetha M., Singh A.K., Asokamani R., Gogia A.K. (2009). Ti based biomaterials, the ultimate choice for orthopaedic implants—A review. Prog. Mater. Sci..

[3] Jiang H. (2018). Enhancement of Titanium Alloy Corrosion Resistance via Anodic Oxidation Treatment. Int. J. Electrochem. Sci..

[4] Moiseyev V.N. (2006). Titanium Alloys: Russian Aircraft and Aerospace Applications.

[5] Leyens C., Peters M. (2003). Titanium and Titanium Alloys: Fundamentals and Applications.

[6] Lütjering G., Williams J.C. (2007). Titanium: With 51 Tables.

[7] Oberwinkler B., Riedler M., Eichlseder W. (2010). Importance of local microstructure for damage tolerant light weight design of Ti–6Al–4V forgings. Int. J. Fatigue.

[8] Knobbe H., Köster P., Christ H.-J., Fritzen C.-P., Riedler M. (2010). Initiation and propagation of short fatigue cracks in forged Ti6Al4V. Procedia Eng..

[9] Fekry A.M., El-Sherif R.M. (2009). Electrochemical corrosion behavior of magnesium and titanium alloys in simulated body fluid. Electrochim. Acta.

[10] Whittaker M. (2015). Titanium Alloys. Metals.

[11] Mountford J.A. Titanium—Properties, Advantages and Applications Solving the Corrosion Problems in Marine Service. Proceedings of the CORROSION.

[12] Al-Mayouf A., Al-Swayih A., Al-Mobarak N., Al-Jabab A. (2004). Corrosion behavior of a new titanium alloy for dental implant applications in fluoride media. Mater. Chem. Phys..

[13] García C., Ceré S., Durán A. (2006). Bioactive coatings deposited on titanium alloys. J. Non-Cryst. Solids.

[14] Sharma A.K. (1992). Anodizing titanium for space applications. Thin Solid Film..

[15] Barjaktarević D.R., Cvijović-Alagić I.L., Dimić I.D., Đokić V.R., Rakin M.P. (2016). Anodization of Ti-based materials for biomedical applications: A review. Metall. Mater. Eng..

[16] Qu Q., Wang L., Chen Y., Li L., He Y., Ding Z. (2014). Corrosion Behavior of Titanium in Artificial Saliva by Lactic Acid. Materials.

[17] Hines J.A., Lutjering G. (1999). Propagation of microcracks at stress amplitudes below the conventional fatigue limit in Ti-6Al-4V. Fatigue Fract. Eng. Mater. Struct..

[18] Sieniawski J., Ziaja W., Kubiak K., Motyk M., Sieniawski J. (2013). Microstructure and Mechanical Properties of High Strength Two-Phase Titanium Alloys. Titanium Alloys—Advances in Properties Control.

[19] Gai X., Bai Y., Li J., Li S., Hou W., Hao Y., Zhang X., Yang R., Misra R.D.K. (2018). Electrochemical behaviour of passive film formed on the surface of Ti-6Al-4V alloys fabricated by electron beam melting. Corros. Sci..

[20] Dadé M., Esin V.A., Nazé L., Sallot P. (2019). Short- and long-term oxidation behaviour of an advanced Ti2AlNb alloy. Corros. Sci..

[21] Chávez-Díaz M., Escudero-Rincón M., Arce-Estrada E., Cabrera-Sierra R. (2017). Effect of the Heat-Treated Ti6Al4V Alloy on the Fibroblastic Cell Response. Materials.

[22] Hussein M., Kumar M., Drew R., Al-Aqeeli N. (2017). Electrochemical Corrosion and In Vitro Bioactivity of Nano-Grained Biomedical Ti-20Nb-13Zr Alloy in a Simulated Body Fluid. Materials.

[23] Zhang L., Duan Y., Gao R., Yang J., Wei K., Tang D., Fu T. (2019). The Effect of Potential on Surface Characteristic and Corrosion Resistance of Anodic Oxide Film Formed on Commercial Pure Titanium at the Potentiodynamic-Aging Mode. Materials.

[24] Reda R., Nofal A., Hussein A.-H. (2013). Effect of Single and Duplex Stage Heat Treatment on the Microstructure and Mechanical Properties of Cast Ti–6Al–4V Alloy. Metallogr. Microstruct. Anal..

[25] El-Bagoury N., Ibrahim K. (2016). Microstructure, Phase Transformations and Mechanical Properties of Solution Treated Bi-Modal Titanium Alloy. Int. J. Eng. Sci. Res. Technol..

[26] Zhao X., Sun S., Wang L., Liu Y., He J., Tu G. (2014). A New Low-Cost β-Type High-Strength Titanium Alloy with Lower Alloying Percentage for Spring Applications. Mater. Trans..

[27] Phukaoluan A., Khantachawana A., Dechkunakorn S., Anuwongnukroh N., Santiwong P., Kajornchaiyakul J. (2011). Effect of Cu and Co Additions on Corrosion Behavior of NiTi Alloys for Orthodontic Applications. Adv. Mater. Res..

[28] Lee C.S., Won J.W., Lee Y., Yeom J.-T., Lee G.Y. (2016). High Temperature Deformation Behavior and Microstructure Evolution of Ti-4Al-4Fe-0.25Si Alloy. Korean J. Met. Mater..

[29] ICDD (1995). PDF 2, Database Sets 1-45.

[30] Lutterotti L., Scardi P. (1990). Simultaneous structure and size–strain refinement by the Rietveld method. J. Appl. Crystallogr..

[31] Lutterotti L. (2010). Total pattern fitting for the combined size–strain–stress–texture determination in thin film diffraction. Nucl. Instrum. Methods Phys. Res. Sect. B Beam Interact. Mater. At..

[32] Belsky A., Hellenbrand! M., Karen. V.L., Luksch P. (2002). New developments in the inorganic crystal structure database (ICSD): Accessibility in support of materials research and design. Acta Crystallogr..

[33] Dollase W.A. (1986). Correction of intensities for preferred orientation in powder diffractometry: Application of the March model. J. Appl. Crystallogr..

[34] Will G., Bellotto M., Parrish W., Hart M. (1988). Crystal structures of quartz and magnesium germanate by profile analysis of synchrotron-radiation high-resolution powder data. J. Appl. Crystallogr..

[35] Cvijović-Alagić I., Cvijović Z., Mitrović S., Panić V., Rakin M. (2011). Wear and corrosion behaviour of Ti–13Nb–13Zr and Ti–6Al–4V alloys in simulated physiological solution. Corros. Sci..

[36] Simsek I., Ozyurek D. (2019). Investigation of the wear and corrosion behaviors of Ti5Al2.5Fe and Ti6Al4V alloys produced by mechanical alloying method in simulated body fluid environment. Mater. Sci. Eng. C.

[37] Mansfeld F. (2005). Tafel slopes and corrosion rates obtained in the pre-Tafel region of polarization curves. Corros. Sci..

[38] Flitt H.J., Schweinsberg D.P. (2005). A guide to polarisation curve interpretation: Deconstruction of experimental curves typical of the Fe/H2O/H+/O2 corrosion system. Corros. Sci..

[39] Flitt H.J., Schweinsberg D.P. (2005). Evaluation of corrosion rate from polarisation curves not exhibiting a Tafel region. Corros. Sci..

[40] Krakowiak S., Darowicki K., Ślepski P. (2005). Impedance of metastable pitting corrosion. J. Electroanal. Chem..

[41] Darowicki K., Krakowiak S., Slepski P. (2004). The time dependence of pit creation impedance spectra. Electrochem. Commun..

[42] Gerengi H., Slepski P., Ozgan E., Kurtay M. (2015). Investigation of corrosion behavior of 6060 and 6082 aluminum alloys under simulated acid rain conditions: Corrosion behavior of 6060 and 6082 Al alloys under acid rain. Mater. Corros..

[43] Blackwood D. (2000). Influence of the space-charge region on electrochemical impedance measurements on passive oxide films on titanium. Electrochim. Acta.

[44] Hamadou L., Aïnouche L., Kadri A., Yahia S.A.A., Benbrahim N. (2013). Electrochemical impedance spectroscopy study of thermally grown oxides exhibiting constant phase element behaviour. Electrochim. Acta.

[45] Gnedenkov S.V., Sinebryukhov S.L. (2005). Electrochemical Impedance Spectroscopy of Oxide Layers on the Titanium Surface. Russ. J. Electrochem..

[46] Cámara O.R., Avalle L.B., Oliva F.Y. (2010). Protein adsorption on titanium dioxide: Effects on double layer and semiconductor space charge region studied by EIS. Electrochim. Acta.

[47] Jorcin J.-B., Orazem M.E., Pébère N., Tribollet B. (2006). CPE analysis by local electrochemical impedance spectroscopy. Electrochim. Acta.

[48] Alqarni N.D., Wysocka J., El-Bagoury N., Ryl J., Amin M.A., Boukherroub R. (2018). Effect of cobalt addition on the corrosion behavior of near equiatomic NiTi shape memory alloy in normal saline solution: Electrochemical and XPS studies. RSC Adv..

[49] Hirschorn B., Orazem M.E., Tribollet B., Vivier V., Frateur I., Musiani M. (2010). Determination of effective capacitance and film thickness from constant-phase-element parameters. Electrochim. Acta.

[50] Krakowiak S., Darowicki K., Slepski P. (2005). Impedance investigation of passive 304 stainless steel in the pit pre-initiation state. Electrochim. Acta.

[51] Dong Z.H., Shi W., Guo X.P. (2011). Initiation and repassivation of pitting corrosion of carbon steel in carbonated concrete pore solution. Corros. Sci..

[52] Amin M.A., Hassan H.H., Abd El Rehim S.S. (2008). On the role of NO2− ions in passivity breakdown of Zn in deaerated neutral sodium nitrite solutions and the effect of some inorganic inhibitors. Electrochim. Acta.

[53] Zakeri M., Naghizadeh M., Nakhaie D., Moayed M.H. (2016). Pit Transition Potential and Repassivation Potential of Stainless Steel in Thiosulfate Solution. J. Electrochem. Soc..

[54] Amin M.A., Abd El-Rehim S.S., Aarão Reis F.D.A., Cole I.S. (2014). Metastable and stable pitting events at zinc passive layer in alkaline solutions. Ionics.

[55] Amin M.A., El-Bagoury N., Mahmoud M.H.H., Hessien M.M., Abd El-Rehim S.S., Wysocka J., Ryl J. (2017). Catalytic impact of alloyed Al on the corrosion behavior of Co_50_Ni_23_Ga_26_Al_1.0_ magnetic shape memory alloy and catalysis applications for efficient electrochemical H _2_ generation. Rsc Adv..

[56] Amin M.A., Fadlallah S.A., Alosaimi G.S. (2014). Activation of Titanium for Synthesis of Supported and Unsupported Metallic Nanoparticles. J. Electrochem. Soc..

[57] Amin M.A., Abd El-Rehim S.S., El-Sherbini E.E.F., Mahmoud S.R., Abbas M.N. (2009). Pitting corrosion studies on Al and Al–Zn alloys in SCN−solutions. Electrochim. Acta.

[58] Scully J.R. (1990). Localized Corrosion of Sputtered Aluminum and Al-0.5% Cu Alloy Thin Films in Aqueous HF Solution. J. Electrochem. Soc..

[59] Pouilleau J., Devilliers D., Garrido F., Durand-Vidal S., Mahé E. (1997). Structure and composition of passive titanium oxide films. Mater. Sci. Eng. B.

[60] Milošev I., Kosec T., Strehblow H.-H. (2008). XPS and EIS study of the passive film formed on orthopaedic Ti–6Al–7Nb alloy in Hank’s physiological solution. Electrochim. Acta.

[61] Wysocka J., Cieslik M., Krakowiak S., Ryl J. (2018). Carboxylic acids as efficient corrosion inhibitors of aluminium alloys in alkaline media. Electrochim. Acta.

[62] Amin M.A., Ahmed E.M., Mostafa N.Y., Alotibi M.M., Darabdhara G., Das M.R., Wysocka J., Ryl J., Abd El-Rehim S.S. (2016). Aluminum Titania Nanoparticle Composites as Nonprecious Catalysts for Efficient Electrochemical Generation of H_2_. ACS Appl. Mater. Interfaces.

[63] Weibin Z., Weidong W., Xueming W., Xinlu C., Dawei Y., Changle S., Liping P., Yuying W., Li B. (2013). The investigation of NbO_2_ and Nb_2_O_5_ electronic structure by XPS, UPS and first principles methods: The investigation of NbO_2_ and Nb_2_O_5_ electronic structure. Surf. Interface Anal..

[64] Kharitonov D.S., Sommertune J., Örnek C., Ryl J., Kurilo I.I., Claesson P.M., Pan J. (2019). Corrosion inhibition of aluminium alloy AA6063-T5 by vanadates: Local surface chemical events elucidated by confocal Raman micro-spectroscopy. Corros. Sci..

[65] Kumar S., Kumar S., Tiwari S., Srivastava S., Srivastava M., Yadav B.K., Kumar S., Tran T.T., Dewan A.K., Mulchandani A. (2015). Biofunctionalized Nanostructured Zirconia for Biomedical Application: A Smart Approach for Oral Cancer Detection. Adv. Sci..

[66] Siuzdak K., Szkoda M., Karczewski J., Ryl J., Darowicki K., Grochowska K. (2017). Fabrication and Significant Photoelectrochemical Activity of Titania Nanotubes Modified with Thin Indium Tin Oxide Film. Acta Metall. Sin. (Engl. Lett.).

[67] Mandrino D., Godec M., Torkar M., Jenko M. (2008). Study of oxide protective layers on stainless steel by AES, EDS and XPS. Surf. Interface Anal..

[68] Wang C., Irfan I., Liu X., Gao Y. (2014). Role of molybdenum oxide for organic electronics: Surface analytical studies. J. Vac. Sci. Technol. Bnanotechnol. Microelectron. Mater. Process. Meas. Phenom..

[69] Liu J., Alfantazi A., Asselin E. (2015). Effects of Temperature and Sulfate on the Pitting Corrosion of Titanium in High-Temperature Chloride Solutions. J. Electrochem. Soc..

