# Supplementary material for: The Influence of Microstructure on the Passive Layer Chemistry and Corrosion Resistance for Some Titanium-Based Alloys
Source: Materials (Basel). 2019 Apr 15;12(8):1233. doi: 10.3390/ma12081233 (PMC6514787; doi:10.3390/ma12081233)
Supplement: Supplementary file 1 [file materials-12-01233-s001.pdf]

Supporting Information

The Influence of Microstructure on the Passive Layer Chemistry and Corrosion Resistance for Some Titanium-Based Alloys

Nader El-Bagoury [1,2], Sameh I. Ahmed [2,4], Ola Ahmed Abu Ali [1], Shimaa El-Hadad [3], Ahmed M. Fallatah [1], G.A.M. Mersal [1,5], Mohamed M. Ibrahim [1,6], Joanna Wysocka [7], Jacek Ryl [7,*], RabahBoukherroub [8] and Mohammed A. Amin [1,9,*]

1 Department of Chemistry, Faculty of Science, Taif University, 21974, Taif, P. O. Box 888, Saudi Arabia; nader_elbagoury@yahoo.com (N.E.); o.abuali@tu.edu.sa (O.A.); a.fallatah.11@hotmail.com (A.M.); gamersal@yahoo.com (G.A.); ibrahim652001@yahoo.com (M.M.);
2 Department of Physics, Faculty of Science, Taif University, 888 Hawiya, Saudi Arabia; sameh@sci.asu.edu.eg
3 Central Metallurgical Research and Development Institute, P.O. Box: 87 Helwan, Cairo, Egypt; shimaamohamad901@yahoo.com
4 Department of Physics, Faculty of Science, Ain Shams University, 11566 Abbassia, Cairo, Egypt
5 Chemistry Department, Faculty of Science, South Valley University, Qena, Egypt
6 Chemistry Department, Faculty of Science, Kafrelsheikh University, 33516, KafrElSheikh, Egypt
7 Department of Electrochemistry, Corrosion and Materials Engineering, Chemical Faculty, Gdansk University of Technology, Narutowicza 11/12, 80-233 Gdansk, Poland; joanna.wer.wysocka@gmail.com
8 Univ. Lille, CNRS, Centrale Lille, ISEN, Univ. Valenciennes, UMR 8520 - IEMN, F-59000 Lille, France; rabah.boukherroub@univ-lille.fr
9 Department of Chemistry, Faculty of Science, Ain Shams University, 11566 Abbassia, Cairo, Egypt
* Correspondence: jacek.ryl@pg.edu.pl (J.R.); maaismail@yahoo.com (M.A.); Tel.: +966 54 570 7507 (J.R.)

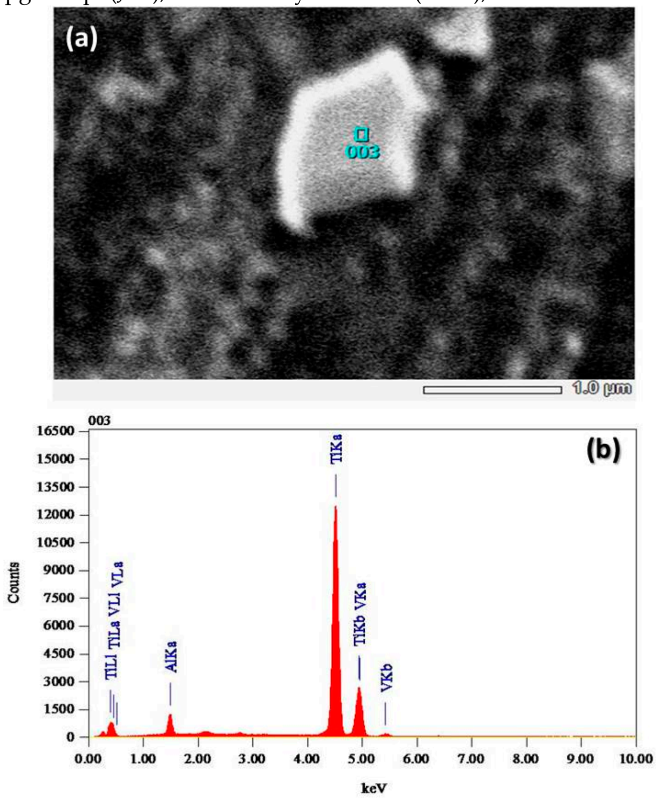

Figure S1 - SEM image (a) and EDS spectrum (b) of α phase in Ti-Al-V alloy.

The EDS spectrum recorded for β phase in the microstructure of Ti-Al-V alloy is depicted in Figure S1 (b). The location of the area of analysis is illustrated in Figure S1 (a). The highest peak in the spectrum belongs to the base metal (Ti), in addition to some other peaks from Al and V alloying elements. Similarly, the analyses of the two phases in other microstructures were accomplished.

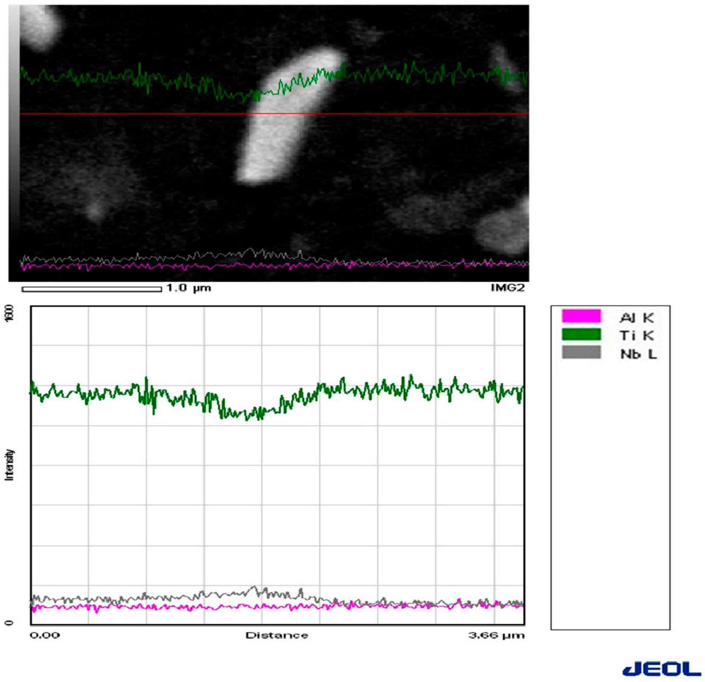

Figure S2 - Line analysis of β phase in TiAlNb alloy.

The line analysis through β phase is shown in Figure S2. The phase β is surrounded by the equiaxed *α* phase in the microstructure of Ti-Al-Nb alloy. The distribution of different alloying elements, Ti, Nb and Al, is illustrated throughout these two phases. The highest line, in green color, represents the distribution of Ti and the grey line depicts that of Nb. Additionally, the third one, in pink color, displays the line analysis of the Al element. Obviously, the β phase has lower Ti and Al contents than the *α* phase, while it exhibits higher amount of Nb compared to *α* phase.

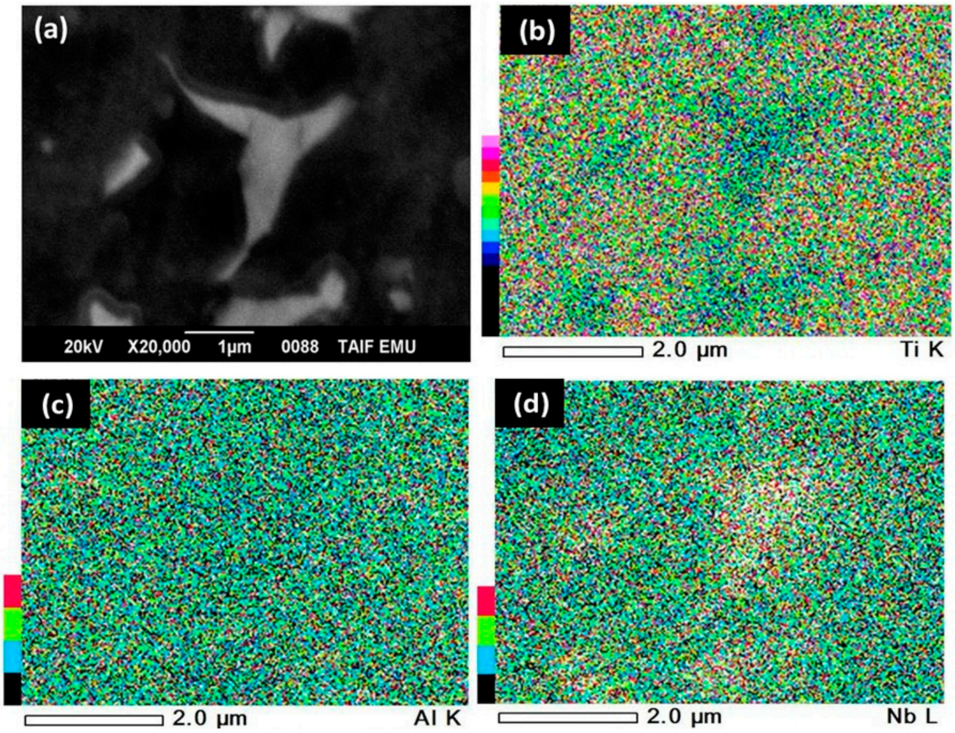

Figure S3 – Microstructure of the Ti-Al-Nb alloy (a), and mapping of Ti (b), Al (c), and Nb (d) alloying elements.

The distribution of alloying elements such as Ti, Al and Nb in Ti-Al-Nb alloy is depicted in Figure S3. Mapping analysis for the bimodal α/β microstructure of Ti-Al-Nb alloy is exhibited in Figure S3 (a), where a clear β phase surrounded by equiaxed α phase is observed at higher magnification, 20000×. Figure S3 (b-d), illustrates the segregation of Ti, Al and Nb alloying elements, respectively. The distribution of these elements between α and β phases is represented by the density of different colors. The rich areas of Ti, Al and Nb elements are decorated by red color, while the poor areas are painted with dark color. In case of Ti element, as it preferably segregated to α phase, as shown in Figure S2, the area of α phase is colored with a combination of red, yellow and pink, however the β phase area, with lower Ti content, has a dark color, see Figure S3 (b). The contrast for the partitioning of Al alloying element between α and β phases is not as clear as in the case of Ti element, Fig. S3 (c). In addition to the distribution of Ti and Al, the segregation of Nb alloying element, with lower percentage, to α phase is represented by a combination of green, blue and black colors, while the area of the β phase with higher Nb content is decorated by a mixture of red and white colors, as shown in Figure S3 (d).

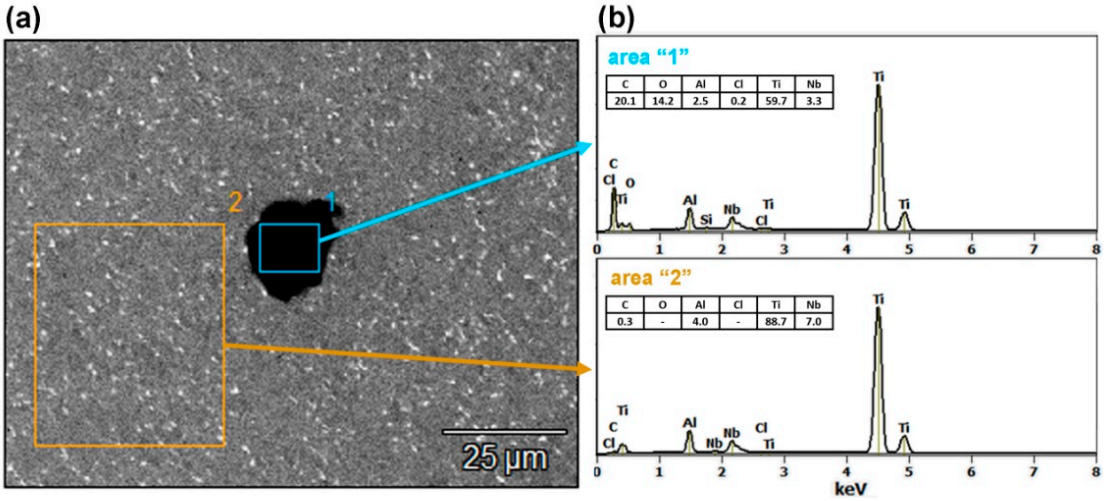

Figure S4 - (a) SEM micrograph with marked areas for EDS analysis, (b) EDS examination at defect and at the surrounding, not corroded area.

Fig. S4 (a) depicts the SEM micrograph and Fig. S4 (b) EDS area analyses for the Ti-6Al-7Nb alloy after one week long exposure to NaCl electrolyte. Area "2" represents adsorbed layer, rich in carbon and oxygen.

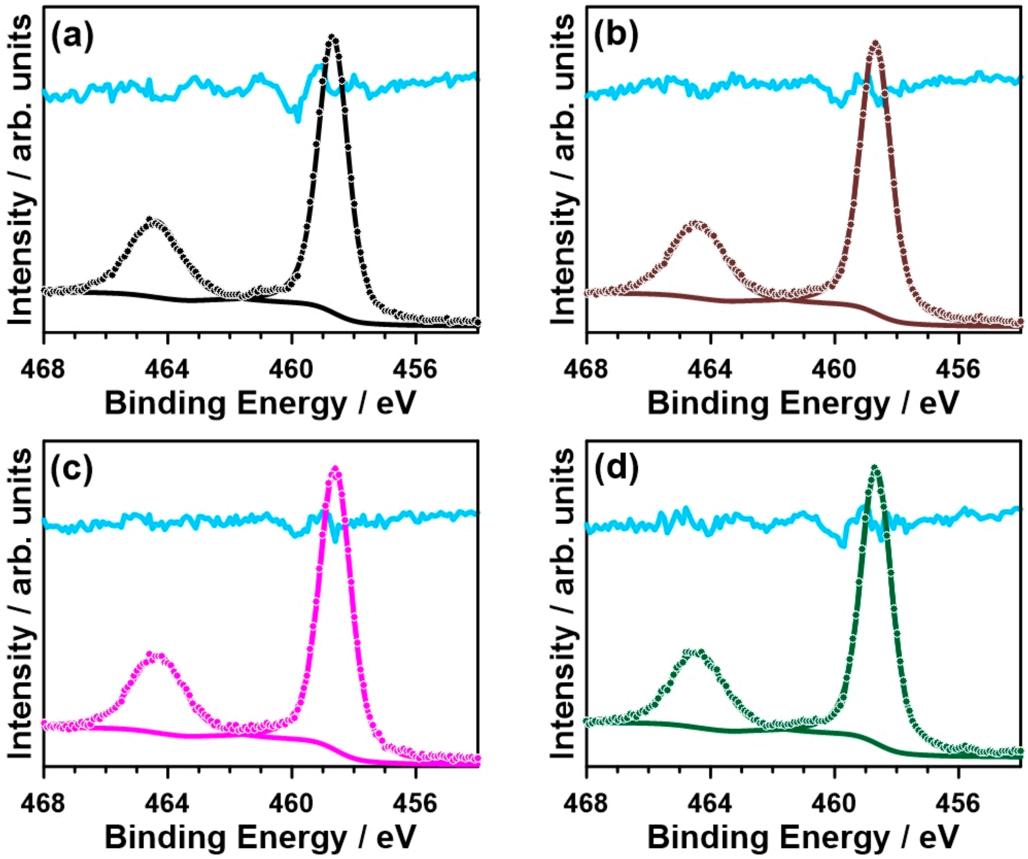

Figure S5 - High-resolution Ti2p XPS spectra with the quality fit (light blue line) for each studied alloy: (a) pure Ti reference, (b) TC21, (c) Ti-6Al-4V, (d) Ti-6Al-7Nb.

The presence of titanium (IV) oxides was confirmed with a peak doublet, $Ti_{2p3/2}$ at 458.6 eV. No other chemical states were required to obtain a good fit during peak deconvolution, as indicated by a light blue line. The FWHM of $Ti_{2p3/2}$ peak ranged between 1.0 (Ti-6Al-4V) and 1.2 eV (TC21).
